# “There is a Place for it”—Practitioners’ Perspectives on Situated Ethical Acceptability of AI Technologies for Mental Health Practice: Qualitative Study

**DOI:** 10.2196/91884

**Published:** 2026-10-19

**Authors:** Abeer Alotaibi, Corina Sas

**Affiliations:** 1 Computing and Communications Lancaster University Lancaster United Kingdom

**Keywords:** mental health, machine learning, practitioners’ perspectives, clinical assessment, diagnosis, treatment, biomedical ethics, HCI, human-AI partnership, explainable AI, human-computer interaction, artificial intelligence

## Abstract

**Background:**

Design research on AI technologies for mental health has expanded rapidly, yet existing work has largely focused on practitioners’ perspectives within isolated therapeutic stages or specific services. As a result, there is limited understanding of how practitioners view the role of AI across the full therapeutic journey, including assessment, diagnosis, and treatment, or how these perspectives can be translated into actionable and ethically grounded design guidance. This lack of stage-specific guidance introduces several risks: (1) the development of AI tools that practitioners may not trust or adopt, (2) heightened ethical concerns, particularly in diagnosis where bias and erosion of human judgment are most likely, and (3) missed opportunities to leverage AI to support ongoing assessment and treatment adaptation.

**Objective:**

This study aimed to explore mental health practitioners’ perspectives on how AI may support or hinder therapeutic practice across all 3 therapeutic stages, with particular attention to their ethical considerations.

**Methods:**

We conducted semistructured interviews with 18 mental health practitioners to explore how AI may support or hinder therapeutic practice across assessment, diagnosis, and treatment. Data were analyzed thematically, and ethical challenges were organized using 4 biomedical principles. This study received approval from the Lancaster University Institutional Ethics Committee (ID 3530).

**Results:**

Findings suggest a stage-specific pattern: practitioners identified valuable roles for AI in supporting clinical assessment and treatment, while expressing strong resistance to autonomous AI diagnosis, which was regarded as the most ethically sensitive stage. Across contexts, AI was seen as supporting and validating, rather than replacing, clinical judgment. Ethical concerns were organized using these principles, with key issues including data validity, inclusivity, privacy, transparency, and explainability. Grounded in these findings, four design directions are proposed: (1) AI-enabled clinical assessment hubs for analyzing multimodal patient data to support transparent decision-making; (2) natural language processing-based tools to enable richer understanding of patients’ lived experiences; (3) new models of trust to support ethically sensitive AI-assisted diagnostic decision-making; and (4) large language model–powered personalized and adaptive mental health support systems that maintain human-AI partnership and preserve practitioners’ central role in care.

**Conclusions:**

This study provides a stage-specific understanding of practitioners’ perspectives on AI use across assessment, diagnosis, and treatment and systematically frames ethical challenges using biomedical principles. The findings translate practitioner insights into actionable design guidance for ethically informed AI-supported mental health practice.

## Introduction

### Project Goals

Design research on AI technologies has seen significant growth, particularly for well-being [1] and mental health [2]. The increased potential for adaptive interactions [3] and personalized interventions [4] has been explored across a range of technologies, from chatbots [5] and virtual assistants [6], which offer scalable solutions for the growing demand for mental health support, to mobile apps and health platforms for understanding user behavior [7], detecting changes in emotional states [8], and delivering personalized mental health interventions [4].

While AI-based technologies have seen significant advancements [2], their adoption in mental health practice has been slow [9]. Understanding the factors that influence acceptance and use in clinical settings is critical. Prior research has explored AI integration from practitioners’ perspectives [4,10-12], but early studies often overlook practical integration into everyday practice.

Recent research has started to address this, for instance by exploring practitioners’ attitudes, learning needs, and the impact of AI on the therapeutic relationship [13-20]. While these studies provide valuable insights, they tend to focus on expert perspectives within a single therapeutic stage or specific service. Less work has comprehensively mapped how practitioners conceptualize AI across the main stages of therapeutic practice, including assessment, diagnosis, and treatment, or systematically translated practitioner perspectives into actionable design suggestions. The 4 principles of biomedical ethics are a well-established framework used often in health ethics [21] and in human-computer interaction (HCI) research [22,23]. More recently, they have also been used to look at AI-related ethical concerns in health care [24,25]; however, little empirical work has mapped ethical concerns across the stages of assessment, diagnosis, and treatment, or used practitioner interviews to do so.

This research is both timely and critical. As AI technologies advance rapidly and become more widely accessible [4], there is increasing pressure to integrate them into mental health services [26]. However, without clear guidance on where and how AI should be deployed across the therapeutic journey, several risks arise [26,27]: (1) the development of tools that practitioners may not trust or adopt, (2) ethical concerns, especially in diagnosis, where bias and loss of human judgment are most likely, and (3) missed opportunities to leverage AI in ongoing assessment and treatment adaptation. To address this gap, we report interviews with 18 mental health practitioners focusing on their perspectives on how AI may better support or hinder therapeutic practice within and across its key stages, with particular attention to ethical aspects. We focused on the following research questions:

RQ1: What are practitioners’ views on the use of AI-based technologies within and across the stages of assessment, diagnosis, and treatment in mental health practice?

RQ2: What stage-specific ethical challenges do practitioners identify, and how do these map onto the 4 principles of biomedical ethics?

Our work makes 3 exploratory contributions. First, we offer a preliminary stage-specific mapping of how a group of UK practitioners conceptualizes AI’s role within and across assessment, diagnosis, and treatment. Second, we organize the ethical concerns raised by participants using biomedical ethics principles as an analytical lens. Third, we translate these practitioner insights into design directions intended to inform and inspire future co-design and evaluation work. These contributions offer an exploratory mapping of practitioners’ perspectives on AI’s role across therapeutic stages and provide a preliminary basis for considering how AI might be responsibly designed and integrated into their practice. Rather than answering the complex question of whether AI should be used in mental health, our work surfaces stage-specific perspectives and ethical tensions that future research and design can build upon.

### Background

Our work builds on research on technologies for mental health, and on the ethics of AI for mental health. Key technologies and ethical aspects from previous research are summarized below.

### AI-Based Technologies for Mental Health

Much work on AI-based mental health has focused on diagnosis and personalized care [2,28,29]. These systems include tools that support assessment and diagnosis, as well as those aimed at treatment or promoting psychological well-being. A range of AI tools have been explored for tracking and intervention, including chatbots [30] and mobile apps [31], each playing a role in assessment, monitoring, and personalized support. However, these technologies have usually been studied on their own, within a single stage of care, and their use across the full therapeutic journey remains underexplored.

AI-powered chatbots have been used to track emotions and offer personalized recommendations, though findings point to ethical challenges and users’ limited AI literacy [32,33]. Woebot showed early promise for stress management and emotional support, but the platform has since shut down, reflecting broader tensions between clinical innovation, regulation, and commercial sustainability [34,35]. Research also shows that transparency alone is often not enough to build user trust [36], pointing to the need for explainable AI and ethical design. More advanced systems include home-based AI therapists using Internet of Things devices and sensors to monitor daily behaviors [37], and digital phenotyping, which collects behavioral data from personal mobile devices to track and infer mental health states [38]. While technically innovative, these approaches raise unresolved ethical concerns around standardization and cultural sensitivity to ensure data collection is effective and respectful across diverse populations. Despite these advances [2], adoption of AI in mental health practice has been slow [9,26]. Understanding what influences acceptance and use in clinical settings is critical.

Recent work has started to address this through studies on professionals’ attitudes toward specific AI technologies [13], learning needs and organizational readiness for AI adoption [14], or surveys of AI use, attitudes, or perceived benefits and harms among practitioners and community members [15]. Another strand of work looked at professionals’ perceptions of large language models (LLMs) for self-care and clinical use [19], with findings indicating that LLMs cannot replace humans due to inappropriate responses and limited relational qualities essential to the therapeutic alliance [20]. Exploration of the broader AI impact on therapeutic relationships [16,17] also highlighted AI’s limitations with respect to empathy and cultural sensitivity, alongside its potential for accessible support [18]. Much such research has explored AI integration in clinical practice from practitioners’ perspectives [4,10-12]; however, it has a predominant focus on a specific service, tool, or therapeutic function.

To conclude, prior work suggests both the potential and challenges of AI-based technologies for mental health, but existing research (as outlined above) has not yet mapped practitioners’ perspectives across the full clinical journey. The present study aims to address this gap by capturing stage-specific practitioner perspectives to inform responsible AI integration.

### Ethics of AI for Mental Health

Another strand of research on mental health technologies has focused on ethics. For instance, Sanches et al [22] used the 4 principles of biomedical ethics [23] in a systematic review of AI-HCI research, finding limited focus on ethics for mental health technologies and highlighting risks such as automated diagnosis for vulnerable users without therapeutic support. Importantly, AI technologies were only minimally addressed in this review, pointing to a gap that subsequent research has begun to address. The ethics of AI for mental health has also been explored in medical research. Some of this work highlights the value of digital phenotyping, with ethical issues related to the privacy of personal data such as social media activity or smartphone usage patterns to identify symptoms [39] and the risk of false positives leading to unnecessary diagnoses [40,41]. Other work has raised a different concern: that replacing human judgment with algorithms risks dehumanizing health care [39,42-44], which is especially problematic in mental health care, where trust and empathy between practitioner and patient are central to treatment [39,45]. Unlike physical health care, where algorithmic decision-making may be more readily accepted, mental health care depends on relational qualities that are difficult to replicate through technology.

Recent work also started to leverage ethical frameworks to explore these gaps, such as the Integrated Ethical Approach for Computational Psychiatry framework [46]: a 5-stage model embedding ethical considerations into AI development for computational psychiatry, which is grounded in 6 values including beneficence, autonomy, justice, privacy, transparency, and scientific integrity. Other scholars applied biomedical ethics principles to evaluate generative AI chatbots [47], showing, for instance, the tensions around autonomy, nonmaleficence, and informed consent. Our work also uses the 4 principles of biomedical ethics framework, albeit with a focus on practitioners’ perspective on the preuse acceptability and the ethical issues of situating AI technologies within their clinical practice. This differs from the common approach through which researchers apply ethical frameworks to AI technologies, albeit with limited emphasis on the specific demands and constraints of clinical practice.

To conclude, research has identified key ethical challenges in AI-based mental health technologies, including harm, privacy, dehumanization, and lack of transparency [39]. However, existing studies tend to examine these concerns in relation to specific tools or populations, without organizing them using an established ethical framework or tracing them across the full therapeutic journey. Understanding stage-specific ethical concerns is essential for ethically grounded design and safe implementation of AI in mental health care [48-50].

## Methods

### Study Design

Our study aims to explore practitioners’ perspectives and interpretation of situated ethical acceptability: their own judgments, based on clinical experience, of whether an AI technology would be ethically acceptable once used within their specific clinical context, rather than an external standard applied to the technology in the abstract. We used this term to refer to preuse acceptability [51], denoting acceptability judged before a technology is actually used. The 4 principles of biomedical ethics [21] were then applied during analysis to organize these situated judgments. We define situated ethical acceptability as the ethical considerations related to the acceptability of technologies such as AI, within the context of clinical practice, so situated, or how it may adapt to its unfolding demands [52].

### Procedure

We conducted individual, online, semistructured interviews with mental health practitioners to understand their perspectives on preuse acceptability of AI technologies and the ethical issues related to their anticipated use in mental health. Thus, the interviews flexibly covered 2 broad areas: (1) practitioners’ views on AI across therapeutic stages, and (2) ethical concerns. Each interview started with a 10-minute presentation (PowerPoint [Microsoft Corp] presentation slides) providing accessible, jargon-free definitions of AI concepts and challenges. For instance, AI was presented as technologies that perform tasks typically associated with human intelligence [53]. We contextualized AI in mental health by outlining its applications in diagnostic processes, predictive analytics, and interventions, including how data may be processed to support diagnosis [54]. We highlighted key challenges associated with AI implementation, including biases in AI algorithms and privacy concerns surrounding the use of sensitive patient data [55]. To help participants reflect on these technologies and their ethical implications, we concluded the presentation with a scenario in which participants were asked to imagine an AI system that analyzes patient input to detect depression symptoms, such as negative language, low mood, hopelessness, and social withdrawal. The system could assess risk levels and offer personalized interventions, including cognitive behavioral therapy (CBT) or mindfulness exercises. This scenario was designed to integrate current AI affordances and ethical concerns, and to provide context for the interview questions. The interview guide and questions used in the interviews are provided in Multimedia Appendix 1.

Following the scenario, participants were asked about their views on preuse acceptability of AI technologies for mental health and anticipated ethical concerns if and when they may become situated in their clinical practice. The focus was on clinical integration and implementation challenges, as well as reflection on the potential benefits and risks, and in particular ethical concerns.

### Participants

Participants were recruited through counseling directories and contacted by email with study details. Only those familiar with AI mental health tools, such as mental health chatbots, and interested in taking part received a consent form and information sheet. This study involved 18 mental health practitioners (16 female and 2 male). While the sample is gender-biased, this reflects the feminization of the mental health profession [56]. Participants were aged 25-55 with an average of 8.4 (SD 6.6) years of experience. Inclusion criteria required participants to be accredited members of the National Association for Counselling and Psychotherapy and to have familiarity with AI technologies. This criterion was stated in the recruitment email, and participants self-selected based on their own assessment of meeting it; those without such familiarity did not proceed to the consent stage. Table 1 presents detailed demographic and educational information.

**Table 1:** Detailed demographic and educational information of participants (n=18).

ID	Gender	Age	Experience (years)	Education
P1	F	27	5	PhD Counselling Psychology
P2	F	26	5	BSc Psychology
P3	F	40	11	PhD Counselling Psychology
P4	F	41	5	MSc Psychodynamic Practice
P5	F	48	15	Diploma Counselling
P6	F	27	4	BSc Psychology
P7	F	27	6	Certificate CBT^a^
P8	F	46	3	Diploma Counselling
P9	F	34	14	Postgraduate Diploma CBT
P10	F	30	6	MSc Counselling/Psychotherapy
P11	F	25	4	BSc Counselling/Psychotherapy
P12	F	30	12	MSc Counselling/Psychotherapy
P13	F	50	2	Diploma Counselling
P14	F	48	30	MSc Integrative Psychotherapy
P15	M	43	10	BSc Psychology
P16	M	45	4	Certificate Counselling
P17	F	55	6	Diploma Counselling
P18	F	44	10	PhD Counselling Psychology

^a^CBT: cognitive behavioral therapy.

### Data Analysis

Interviews were fully transcribed and analyzed using ATLAS.ti software [57], following a hybrid thematic analysis approach [58], combining deductive and inductive coding. This process resulted in over 1130 codes across the 18 interviews. The deductive codes were informed by health research on the 3 main stages of psychotherapy (assessment, diagnosis, and treatment) and the 4 principles of biomedical ethics (nonmaleficence, beneficence, justice, and autonomy) [21].

Inductive codes were generated to capture the richness of interview data. The coding process followed a structured, iterative process for over nearly 3 months through ongoing conversations within the research team. This team was drawn from complementary expertise in AI-HCI in mental health and HCI and ethics of mental health. The coding process was initially inductive, with deductive codes being generated as links to relevant theories, such as ethical principles, were identified. The first author conducted 1 pass through part of the interviews, generating the initial set of codes. The second author independently reviewed the codes, and the team discussed their definitions, distinctiveness, and overall organization. Main changes at this stage included splitting broad codes such as the one on privacy, including “concerns around data misuse,” “lack of transparency in AI decisions,” and “patients’ inability to control their own data,” into separate distinct codes of privacy in relation to the 4 principles of biomedical ethics [21], namely privacy and confidentiality under autonomy, transparency and explainability under beneficence, data validity under nonmaleficence, and inclusivity and accessibility under justice.

Disagreements between the 2 coders were resolved through a consensus-based approach, commonly applied in HCI research [59], and once consensus was reached on the structure and definitions of all codes, the coding scheme was applied for the analysis of the remaining interviews. The final set of codes included both inductively generated codes as subcodes under the main deductive codes. For example, within the treatment stage, inductively generated subcodes include the following: recommending personalized interventions, ongoing clinical assessment, delivering timely interventions, monitoring effectiveness, proactively adapting interventions, memorializing the therapeutic journey, and supporting patient engagement.

The code frequencies (Table 2) indicate how frequently topics such as views on the use of AI technologies across therapy stages and their ethical challenges were reported by participants. Such prevalence, however, is not a measure of salience. For the latter, we considered instead experts’ detailed accounts and their specific emphasis on specific codes, as reflected in the quotes reported in the Results section.

**Table 2:** Distribution of main codes and their subcodes related to practitioners’ views of AI technologies for mental health and their ethical challenges. The table shows the frequency of each main code and subcode within the total number of codes (n=1130).

Code			Values, n (%)
**Practitioners’ views of AI for mental health (n=981)**			
	AI value for clinical assessment	375 (38.26)	
	AI value for diagnosis	63 (6.43)	
	AI value for treatment	543 (55.31)	
**Ethical challenges (n=149)**			
	Nonmaleficence	28 (18.79)	
	Beneficence	44 (29.53)	
	Justice	39 (26.17)	
	Autonomy	38 (25.50)	

### Ethical Considerations

This study was approved by the Lancaster University Institutional Ethics Committee (approval number 3530). Participants were recruited through counseling directories and contacted by email with study details. Only practitioners who were familiar with AI mental health tools and interested in taking part received the participant information sheet and consent form, and informed consent was obtained from all participants before the interviews. All interviews were conducted by the first author, who has expertise in AI-HCI for mental health, online via Microsoft Teams. Interviews were audio-recorded with participants' consent and fully transcribed. To protect participants' privacy, they are identified only by anonymized participant codes (P1-P18) in the paper, and no identifying information is reported. Each interview lasted approximately 1 hour, ranging from 48 to 65 minutes. Participants were compensated with £40 (approximately US $54) for their time.

## Results

### Overview

This section highlights practitioners’ views on AI technologies for mental health across 3 key stages of clinical practice, and their ethical challenges reflected in the 4 principles of biomedical ethics. As our findings are from a study with a sample of UK mental health practitioners, the insights presented here are intended as a starting point for understanding how AI may be perceived and integrated in practice, rather than a complete picture of the field.

### Practitioners’ Views on AI Technologies for Mental Health

Our findings provide insights into how this group of mental health practitioners perceives AI technologies across the main stages of psychotherapy and counseling. Treatment and clinical assessment were discussed more frequently than diagnosis across participants. These frequencies are presented to describe the distribution of themes within the dataset rather than their relative importance. Although diagnosis appeared less frequently, participants who discussed this stage consistently emphasized concerns regarding autonomous AI decision-making, dehumanization, and the need for practitioner oversight. These qualitative accounts, rather than coding frequency itself, informed our interpretation of diagnosis as an ethically sensitive stage.

### AI Value for Clinical Assessment

Clinical assessment consists of gathering information to identify patients’ challenges, problematic behaviors, and symptoms, usually through observation and valid assessment questionnaires or tests [60]. Findings suggest that participants perceived value in AI technologies for gathering and analyzing data captured through self-reports on personal information such as patients’ age, gender, physical health, mental health, and therapy history, childhood experiences (P2), family background, lifestyle, specific traumas, work-related issues, financial issues, or friendship and family relationship dynamics (13/18). Other types of data can be gathered through patients’ entries in digital diaries to track moods (5/18), thoughts, and behaviors, as well as links between these (3/18). Findings also suggest that practitioners perceived AI as potentially able to automate note-taking and documentation tasks, particularly for recording symptoms (3/18). In addition to self-reports, practitioners also discussed the added value of other data types such as voice input (2/18), body language (4/18), biosignals, that is, heart rate (P17), as well as contextual analysis of diary entries (4/18).

### AI Value for Diagnosis

Participants expressed resistance to autonomous AI-based diagnosis without practitioner oversight, describing concerns about the impact on patients when diagnosis is delivered by technology alone:

Ethically as well, I would say [...] making sure that it’s client centered and almost it doesn’t dehumanize them, and it doesn’t just put it into simply, you know, depressed, really high risk, and it’s it just takes the nuances out of it.

P11

On the other hand, participants expressed that AI might add value for diagnosis, but merely to validate and support practitioners’ decision-making: “the clinician could then look at the data, look at the sort of diagnosis that the technology thinks that [patient] has, and then work off of that” (P7).

### AI Value for Treatment

Treatment stage involves the development of the most appropriate personalized and detailed treatment plan (7/18) informed by the clinical assessment and diagnosed condition. Participants reported a range of topics reflecting the potential value of AI within this stage, which we clustered under recommending personalized interventions, ongoing clinical assessment, delivering timely interventions, monitoring and adapting interventions, memorializing therapeutic journey, and increasing patients’ engagement.

### Recommending Personalized Interventions

Participants perceived potential value in AI for generating effective treatment plans: “using the diagnostic criteria and [...] information given by client [AI] will use that to make [...] a virtual diagnosis [...] and then based on that, come up with a [treatment] plan” (P3). Such plans, however, should be specific, with low-level details for each patient rather than generic information (P15). They also mentioned the value of using lists of valid interventions and microinterventions for main mental health conditions which AI technologies could leverage in order to recommend personalized interventions (P7). Participants also highlighted the value of clear communication of the AI-suggested intervention and its goals, so that both practitioners and patients can judge its value and “agreed [that] this is what the client wants [and] needs” (P10).

### Ongoing Clinical Assessment

Clinical assessment is not merely a one-off activity at the start of therapy, but an ongoing process aimed at ensuring that the progress of the therapeutic plan is as expected [60]. While traditionally, clinical assessment is completed within the therapeutic sessions, ongoing clinical assessment is recurrent throughout the entire therapy journey. Thus, some participants acknowledged the value of AI technologies for tracking patients’ symptoms and their changes over time (2/18). They particularly emphasized AI-based analysis (6/18) to inform AI-based decisions (8/18). Practitioners perceived that this could support a better understanding of patients’ experiences and a potentially more robust ongoing assessment (P15), while reducing some of the practitioners’ burden:

I’d say really positive because [AI] helps in between sessions when I’m working with them and also when we finish our counselling together, there are things they can continue to use.

P7

Additional value of AI technologies consists of extracting nuanced meanings for deeper understanding of patients and their conditions (P12), for instance through keyword and phrase detection (P7) or through the classification of important data (6/18). Key here is the swift analysis of diary entries (P15) with a focus on changes in patients’ data (2/18) to identify risk factors, red flags, triggers, and changes in symptoms (5/18).

[If AI could] gather data to get [a] clear understanding of depression [...] would be very helpful [...] For example, monitoring a client in crisis [...] if they haven’t got mental health support, there is potentially a place for [AI] if it is carefully policed.

P17

Participants also noted that such risk analysis (5/18) could complement as well as validate or improve the accuracy of practitioners’ own assessment (6/18) performed through traditional time-consuming methods such as observation and conversations (3/18).

### Delivering Timely Interventions

Practitioners indicated that based on ongoing clinical assessment, AI technologies could suggest ongoing interventions outside therapy sessions (8/18):

In between sessions, it would be really helpful, especially for an approach like Cognitive Behavioral Therapy (CBT), to see certain patterns [based on patients] journaling about that.

P7

Practitioners expressed the view that AI technologies could recommend specific interventions to promptly meet patients’ needs in the moment. This benefit of AI technologies has been particularly confirmed for patients with worsening symptoms:

It would be useful in terms of if suddenly somebody [...] seem to have deteriorated outside of the norm for that person [...]it would give you something to talk to them about it.

P4

### Monitoring the Effectiveness of Interventions

While ongoing clinical assessment also enables the monitoring of treatment’s progress and its effectiveness, practitioners anticipated that AI technologies could track changes in symptoms to evaluate and report such effectiveness (7/18). In particular, the identification of symptom and behavior patterns between therapy sessions (3/18) emerged as a notable strength, providing practitioners with valuable insights into the effectiveness of interventions.

### Proactively Adapting the Interventions

Ongoing clinical assessment can also reflect significant changes in patients’ risk factors (9/18), which require adjustment of the treatment plan. Participants mentioned the value of AI technologies to discover these factors and predict their impact on the worsening of the symptoms in order to proactively recommend adaptive interventions (4/18). A practitioner expressed optimism that AI-based risk analysis could complement traditional risk assessment methods relying on observational methods during therapeutic sessions (P1).

### Memorializing Therapeutic Journey

Both counseling and psychotherapy take time and commitment, so monitoring patients’ progress over time through ongoing clinical assessment becomes important. This is, however, taxing, both for patients and practitioners, and rarely provided in visual formats (3/18). The value of AI technologies for retrospective evaluation of interventions, particularly through a timeline approach, was highlighted as a strength.

To have something in place at the beginning [as] part of ongoing intervention is really helpful [so] I’m able to look back and kind of provide some kind of timeline to look at how long they’ve been depressed.

P1

### Supporting Patients’ Engagement

An important outcome is participants’ mention of the anticipated impact of AI-based technologies on patients’ engagement in the therapeutic journey through increased ownership and accountability. More specifically, the manual data entry in online journals can extend beyond therapy sessions, fostering a sense of ownership, while also reducing practitioners’ need for extensive documentation (6/18). Practitioners viewed AI-based pattern detection as a factor that could strengthen patients’ accountability by supporting awareness and introspection about the links between specific behaviors and well-being or mental ill health symptoms (6/18). For instance, this illustrative quote points out the issue of accountability and ownership:

I would try and get clients to [journal] but I think it would be really helpful to have that shared with the clinician [...] it holds the client a bit more accountable [...] helpful to [...] notice certain patterns.

P7

Participants further suggested incentives for engagement by incorporating, for instance, visual elements and gamified features such as growing trees to engage and support clients in their mental health journey (3/18).

Grounded in the above benefits of AI technologies, participants identified subsequent ones such as reducing clinicians’ time (P7) and cost (P1), contributing to enhanced therapeutic outcomes (P1) and operational efficiency of therapeutic practices. Practitioners anticipated this could partly address practitioners’ shortage (2/18), which in turn can lead to increased patient admission rates (P1) and reduced waiting time (P17). Table 3 presents a summary of practitioners’ perspectives on how AI technologies may support mental health practice across assessment, diagnosis, and treatment stages.

**Table 3:** Summary of practitioners’ perspectives on how AI technologies may support mental health practice within and across the main therapeutic stages of assessment, diagnosis, and treatment. The content within each cell reflects themes derived from the interview data.

Category	Assessment	Diagnosis	Treatment
Primary goal	Gather and organize comprehensive patient information	Validate and support practitioner diagnostic decisions	Deliver continuous personalized therapeutic support
Data role	Collection hub—passive gathering	Analysis layer—pattern matching	Intervention system—real-time response
AI autonomy	High autonomy in data processing with practitioner oversight	Low autonomy—requires practitioner validation	Medium autonomy—delivers within approved protocols
Ethical sensitivity	Concerns related to data privacy during collection and storage	Highest risk—dehumanization, harm if autonomous	Moderate risk—engagement vs monitoring balance
Temporal scope	One-time or periodic baseline	Single event decision point	Continuous monitoring throughout therapy
Core AI capabilities	Data aggregation, multimodal input processing, pattern extraction, documentation automation	Symptom pattern matching, diagnostic criteria mapping, decision support, validation	Personalization, real-time monitoring, adaptive algorithms, timeline visualization, risk prediction
Expected outputs	Structured patient profile, symptom summary, documentation	Diagnostic suggestions, supporting evidence, confidence levels	Treatment recommendations, intervention timing, progress visuals, adaptation alerts
Practitioner role	Guide and review—oversee data gathering process	Decision-maker—validate all AI suggestions before use	Supervisor—monitor AI delivery, adjust protocols as needed
Key success factors	Accuracy of data capture, multimodal integration, user ease, privacy protection	Explainability, transparency, validation support, clinical accuracy	Timing, personalization, engagement, effectiveness measurement, adaptability
Practitioner benefits	Reduced documentation time, comprehensive data access, consistency	Decision confidence, pattern visibility, reduced cognitive load	Extended reach (between sessions), proactive intervention, efficiency gains, better outcomes

### Ethical Challenges

Practitioners suggest key ethical concerns grouped under the 4 biomedical ethics principles proposed by Beauchamp and Childress [21] and previously applied in HCI mental health research [22]. These principles offer a valuable analytical lens because they bridge the gap between medical ethics and HCI research on mental health technologies, providing a theoretically grounded and clinically recognized framework for examining ethical concerns at the intersection of AI and therapeutic practice [22,61]. Following Beauchamp and Childress [21], nonmaleficence refers to a clinician’s duty to avoid harm to the patient; beneficence captures the clinician’s duty to act in the patient’s best interest; justice is about fair distribution of benefits and burdens in health care across all people; while autonomy refers to respect for the patient’s right to make informed decisions. These principles have informed the organization of the ethical concerns practitioners raised about AI integration across therapeutic stages.

### Nonmaleficence Principle: Limited Data, Validity of AI-Based Decisions

From the lens of the nonmaleficence principle, practitioners expressed concerns about the limited data used for AI-based decisions and the validity of AI decisions (7/18). The issue of limited data has 2 dimensions. First, current technologies capture data that are insufficient to fully understand the richness of patients’ experiences and their broader context. For example, 1 participant mentioned:

Someone can even say I’m happy, but they’re crying, you know? But then their crying doesn’t look like happy tears. It looks like sadness tears. These are the things that, if you write them down and put them into the system, it will not be able to detect that.

P3

Second, the data may not be accurate due to technology or patients’ errors, including limited memory, awareness, or introspection:

Is the client gonna be fully truthful? or are they aware of some things they might write down on there? [...] they might think something about themselves that isn’t necessarily true.

P11

Practitioners also described the challenges of understanding deeper meaning (P5) from data such as suicide detection, or of differentiating between similar symptoms (P13), and questioned the potential of AI for such analysis.

Participants mentioned that limited data and AI’s ability to probe for meaning can be harmful when used to make predictions, identify risk factors, make diagnosis decisions, or recommendations for interventions (9/18). Some participants also raised concerns about the potential loss of human intuition in the therapeutic process due to the integration of AI (5/18). As mentioned by 1 participant*:* “These could be very desperate people [...] and those interventions [...] there is the potential that they won’t even scratch the surface of helping these people” (P16). Participants also suggested that AI-based interventions tend to support behavioral interventions (P1), which may not align with relational or person-centered approaches (2/18).

To mitigate the risk of dehumanizing or oversimplifying the complex therapeutic processes due to problematic data and AI-based decisions, participants suggested the importance of integrating AI technologies within therapeutic practice, so that they are used only with practitioners’ oversight:

I think it needs to be carefully managed and monitored because you don’t know where it’s taking them. I think there is a place for it, but I think it very much depends on the needs of the client and the severity of their condition. I think there are significant risks associated with not having any human intervention to understand where that client is and what the AI is telling them.

P17

### Beneficence Principle: Support Assessment and Rapport

The principle of beneficence concerns the clinician’s duty to act in the patient’s best interest [21]. Practitioners identified current limitations in data and tools as key barriers. They described ongoing clinical assessment as limited by inadequate note-taking tools (3/18) and fragmented data, noting that facial expression, speech, and posture are essential to their work (3/18), and that their absence can lead to emotional disconnect (4/18). Missed emotional cues or incomplete data can therefore result in delayed or inappropriate interventions, directly working against the patient’s best interest.

Practitioners suggested that AI could help address these gaps by capturing richer patient data and enabling more in-depth analysis through categorization tools integrated in a centralized assessment platform (3/18). Such tools should track how symptoms change over time (3/18), and across domains such as work and relationships (2/18):

Anxiety has various categories [...] so if [AI] came up with social anxiety, I think I’d find that more useful [...] I would be more interested in [AI] that had got to the bottom of what kind of anxiety [also] interesting to see [if AI] could thematically analyze [data] so whether it’s about family stuff, work stuff and what is the context of some of these diary entries alongside almost the harder stuff such as length [or] type of suffering [...] what’s it related to?

P15

### Justice Principle: Inclusive and Accessible AI Technologies

The ethical principle of justice relates to fair distribution of benefits and costs/risks for all people regardless of race, gender, or class. Aligned with this principle, participants highlighted the challenge of inclusive AI technologies. This includes ensuring that AI systems are accessible to diverse populations. Other concerns relate to the costs of such technologies, which patients may not be able to afford. Participants expressed additional concerns about the cognitive load that AI technologies may involve (3/18), as well as for practitioners who may need to engage with large amounts of data (2/18).

To address these challenges, practitioners suggested using AI training data from diverse populations and designing interfaces that match users’ accessibility needs and preferred interaction (6/18). Few participants called for making these technologies more cost-accessible (3/18), while others highlighted the need for increased AI literacy (3/18).

### Autonomy Principle: Privacy, Transparency, and Explainability

The ethical principle of autonomy reflects respect for people’s decisions, and its concerns relate to privacy and confidentiality, as well as transparency of AI-based decisions. Some participants highlighted privacy and security concerns (Textbox 1: summary of ethical challenges of AI technologies for mental health across the 4 biomedical ethics principles: nonmaleficence, beneficence, justice, and autonomy) for all patients’ personal and sensitive data on mobile apps (4/18) or online technology, particularly for the most vulnerable ones such as children (3/18). They expressed apprehension about how personal data is stored, secured, and handled, as well as potential data breaches in AI technologies (12/18). These privacy challenges extend to ensuring the confidentiality of the practitioner-patient relationship (P2).

**Ethical challenges of AI technologies for mental health across the 4 biomedical ethics principles: nonmaleficence, beneficence, justice, and autonomy.**

1. Nonmaleficence

**Limited data–Clinical assessment**

- Data insufficient to understand the richness of patient experiences.
- Cannot detect contextual cues (eg, happy vs sad tears).
- Nonverbal signals and bodily cues missing from records.

**Data validity and accuracy–Clinical assessment, treatment**

- Patient errors in self-reporting due to limited memory.
- Patients provide inaccurate assessments.
- Harm when AI identifies risks or recommends interventions on flawed data.

**Loss of human intuition–Diagnosis, treatment**

- Reliance on AI may diminish practitioners’ clinical judgment.
- AI interventions may not address deeper therapeutic needs.

**Therapeutic approach misalignment–Treatment**

- AI supports behavior interventions primarily.

2. Beneficence

**Limited assessment tools–All stages**

- Ongoing clinical assessment supported by limited tools.
- Fragmented data collection across sessions.
- Difficult to track emotional experiences over time.

**Absence of bodily cues–Clinical assessment**

- Absence of bodily cues leads to emotional disconnect.
- Practitioners cannot access rich nonverbal data.

**Limited data analysis–Clinical assessment**

- Systems cannot probe for deeper meaning in symptoms.
- Difficulty differentiating between similar symptoms.

3. Justice

**Accessibility and inclusivity–All stages**

- AI systems are not accessible to diverse populations.
- Technology not adapted to different user needs.
- Risk of excluding marginalized groups.

**Cost and financial barriers–All stages**

- High costs inaccessible to many patients.
- Unequal distribution of benefits and support.

**Cognitive load and data complexity–All stages**

- AI interfaces impose high cognitive load on patients.
- Practitioners struggle managing large data amounts.
- Limited AI literacy in user populations.

**Data diversity and bias–Diagnosis**

- Training data not representative of diverse populations.
- AI models may not work equitably across groups.

4. Autonomy

**Privacy and security risks – Clinical assessment, treatment**

- Potential data breaches exposing mental health information and sensitive data.
- Special concerns for vulnerable populations such as children.

**Transparency and explainability – Diagnosis, treatment**

- Lack of clear rationale for AI decisions.
- Practitioners unable to understand AI recommendations.
- Black-box nature reduces informed consent.

**Limited informed consent – Clinical assessment, treatment**

- Patients lack understanding of data usage and transparency.

To mitigate these challenges, participants suggested security features for AI-based mental health technologies, including authentication through coded data such as anonymized ID rather than personal information (3/18), password protection (4/18), and avoiding recording of any identifiable data such as emails (P12). With respect to mobile apps, several participants (4/18) highlighted the need for increased transparency in app design, for instance through clear communication on data usage (P2). Other suggestions were aimed at protecting the practitioners’ entered data, for example through secure note-taking or documentation functionalities, as well as additional functionalities for supervision notes to maintain confidentiality and professionalism (11/18). Findings also suggest awareness of the challenges of AI-based decisions and the need for their clear rationale and layperson explanations (3/18), which in turn could increase practitioners’ AI literacy: “I would want to see how [AI] reached that recommendation [...] it would be helpful to see some kind of pathway [how] did it get there? Oh, I can see, there was that symptom” (P1).

## Discussion

### Principal Findings and Comparison With Prior Work

With regard to RQ1, findings suggest that practitioners’ acceptance of AI is stage-specific. For assessment, they wanted AI to be used for gathering and organizing rich patient information, such as self-reported background data, mood and behavior tracking through digital diaries, and automating note-taking, in order to build a more complete picture of each patient over time. This aligns with prior calls for richer, multimodal input data in mental health apps [62].

Such AI functionalities were framed as extending, rather than replacing, clinical judgment, which matters in light of prior findings that AI chatbots are limited by users’ AI literacy [32,33]. As expert practitioners, our participants were in a strong position to identify where AI may add value to their practice or not, rather than accepting or rejecting AI as a whole. For diagnosis, practitioners wanted AI to be used only to validate and support their own diagnostic judgment, rather than to generate a diagnosis independently, and they expressed concerns that AI-led diagnosis could strip away the nuance of a patient’s presentation and reduce them to a label rather than a person. This aligns with prior HCI research cautioning against fully autonomous AI-based diagnosis without practitioner oversight [22] and extends prior work that examined AI adoption without distinguishing between therapeutic stages [63]. For treatment, practitioners wanted AI to be used for recommending personalized interventions, delivering timely support between sessions, and monitoring and adapting treatment plans as patients’ symptoms changed, which echoes prior calls for explainability in AI-supported care [4].

This preference for AI to become embedded under practitioner oversight also helps explain why earlier tools struggled to remain adopted: Woebot showed early promise but ultimately ceased operations, reflecting the tension between clinical innovation and sustainable integration into everyday practice [34,35]. More broadly, our stage-specific findings offer one explanation for why adoption of AI-based mental health technologies, including advanced tools such as home-based AI therapists and digital phenotyping [37,38], has remained slow [9,26]. Thus, we argue that practitioners are not resistant to AI itself, but to AI applied without regard to the specific therapeutic stage. Prior studies on practitioners’ attitudes and adoption readiness [13-17,19,20] have largely examined a single tool or service; our findings begin to address this gap by showing that acceptance depends on which stage of the therapeutic journey AI is applied to.

With regard to RQ2, we explored the ethical aspects of AI technologies for mental health, and our findings highlight concerns under each of the 4 principles of biomedical ethics [21]. We also build on prior work [63] to organize these concerns according to the 4 principles, rather than treating them in isolation. Under nonmaleficence, we have concerns centered on insufficient or inaccurate patient data, and AI’s limited ability to capture deep or nuanced meaning. This risks leading to invalid or harmful decisions if used without practitioner oversight, confirming and extending prior concerns about unsupported diagnostic labeling [22]. Under beneficence, we have those concerns centered on the loss of emotional cues, such as facial expression, posture, and tone, in AI-mediated data collection, which can hinder empathy and compromise the clinician’s capacity to act in the patient’s best interest. This echoes previous concerns that algorithmic decision-making struggles to replicate the relational qualities central to mental health care [39]. Under justice, practitioners raised concerns about the accessibility and inclusivity of AI technologies, including cost, cognitive load, and the risk that systems trained on nondiverse data may not serve all populations equally.

Thus, our findings extend prior justice-related critiques of AI-based mental health apps [33]. Under autonomy, we group those concerns centered on the privacy and security of sensitive patient data and the confidentiality of practitioners’ notes, alongside the need for transparency and explainability in AI-based decisions. These are aligned with similar tensions identified in evaluations of generative AI chatbots [47]. We also note that while the ethical concerns are organized under the 4 principles of biomedical ethics as a primarily descriptive tool, our analysis leverages findings on the outstanding ethical concerns in order to inform actionable design knowledge of how such concerns may be addressed by future AI technologies for mental health clinical practice.

### Implications for Design

Our findings informed 4 implications for design research described below. We propose four design directions: (1) clinical assessment hubs using AI to analyze multimodal patient data for transparent decision-making; (2) natural language processing (NLP)–AI tools for richer understanding of patients’ experiences; (3) new models of trust in AI-based diagnosis decisions; and (4) LLM-powered personalized, adaptive mental health support systems that maintain human-AI partnership and practitioners’ central role (Figure 1).

**Figure 1:**
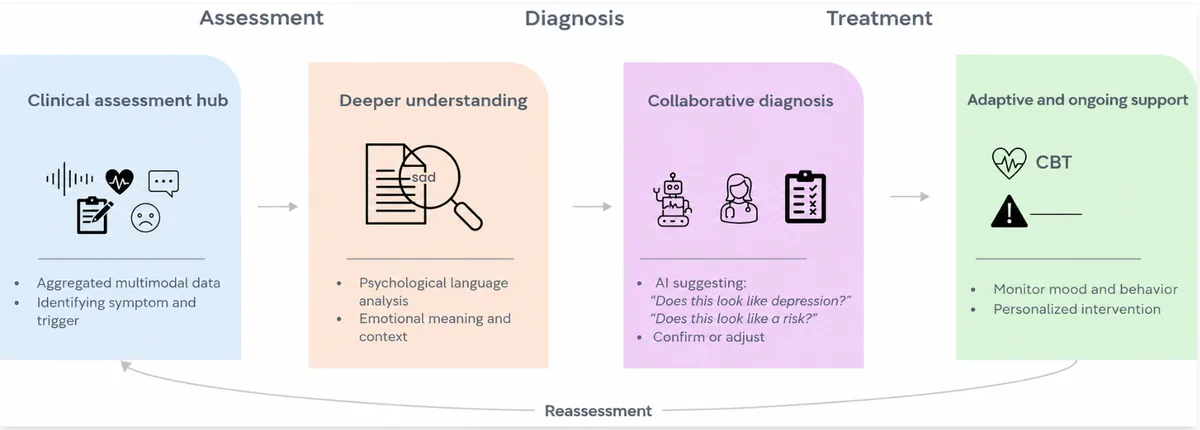
This figure presents our design implications for mental health technologies. It illustrates how AI systems can be designed to ethically and transparently support practitioners within and across assessment, diagnosis, and treatment stages. CBT: cognitive behavioral therapy.

### AI Data and Models as Resource for Design: Clinical Assessment Hub

This design implication is grounded in our findings, where practitioners identified clinical assessment as an important stage for AI and highlighted two specific challenges aligned with our ethical framework: (1) beneficence: current assessment relies on fragmented, limited data and manual note-taking, missing important patterns over time; and (2) nonmaleficence: the need to avoid oversimplifying complex patient experiences through richer data categorization. These principles point to a transparent, multimodal hub that combines richer data with explainability and practitioner oversight, rather than data collection alone.

While most practitioners described wanting AI to capture body language and diary entries alongside self-report data, some also suggested voice input and biosignals as additional data types worth exploring in future design work. To address these, we propose a Clinical Assessment Hub, a centralized AI-powered platform that integrates multimodal data (text, voice, facial expression, biosignals) to support practitioners in identifying symptoms, risk factors, and triggers through transparent, explainable, and practitioner-supervised decision-making. This is inspired by practitioners’ expressed need for more robust, centralized assessment tools and reflects the principles of beneficence and nonmaleficence by maintaining human oversight and transparency to minimize potential harm.

This design direction is directly motivated by practitioners’ accounts. Participants described the limitations of existing note-taking tools, the absence of bodily cues in remote sessions, and the clinical value of tracking how symptoms change over time across domains such as work and relationships. These practitioner-identified gaps point toward a centralized hub as a design response, rather than a fully specified technical solution. These outcomes open up design opportunities for positioning data and AI models as exciting resources for interaction design. For this, we can draw inspiration from emerging work on data practices [64], or comparative exploration of AI models for predicting conditions [7]. For instance, the study by Chen et al [65] has shown the value of supervised learning for differentiating specific mental conditions, unsupervised learning for identifying condition subtypes, and regression models for predicting changes in symptoms. We draw on these examples to illustrate the kinds of analytical capabilities that could be explored in future design and evaluation work, in response to the multimodal data needs articulated by our practitioners.

We suggest the value of such a tailored approach to AI models for analyzing integrated multimodal data to support the identification of symptoms, risk factors, and triggers, and their change over time, both actual and predicted. This could draw on AI models for analyzing speech, facial expressions, and contextual information [66-68], as well as emerging approaches such as changepoint detection [69] and predictive analysis models [70]. The integration of multimodal data from both patients and practitioners, as described by participants, can lay the foundation for such a hub as a practitioner-centered tool, one that future co-design and implementation studies should further develop and evaluate.

### Innovative NLP-AI Tools for Deeper Meaning-Making

This design implication addresses a key nonmaleficence concern identified by practitioners: AI’s inability to probe deeper meaning in patient data, including challenges around suicide detection, symptom differentiation, and thematic analysis of diary entries. These concerns arose directly from participants’ accounts. Practitioners described how a patient might say they are happy while displaying visible sadness and questioned whether AI could differentiate between similar symptoms or detect deeper emotional meaning in self-reported text. They also highlighted the challenge of thematic categorization. For example, whether AI could distinguish social anxiety from other forms of anxiety, or identify whether diary entries relate to family, work, or other life domains. These concerns highlight that limited data validity could lead to misdiagnosis, particularly for conditions such as depression, where subtle emotional and cognitive patterns are clinically critical. Rather than simply recommending more advanced NLP models, nonmaleficence points specifically to the need for explainable NLP systems that minimize clinical harm through contextual, transparent interpretation, rather than more accurate but opaque prediction.

To address this, future design opportunities could explore transformer-based NLP models capable of contextual interpretation of patient data while reducing the risk of clinically harmful misinterpretation. Models such as ClinicalBERT [71] and MentalBERT [72] are potentially relevant here. For example, ClinicalBERT was pretrained on clinical notes from electronic health records, giving it familiarity with medical language and symptom descriptions that general-purpose language models lack; while MentalBERT was specifically pretrained on mental health-related text from social media and clinical sources, making it better suited to the informal, emotionally laden language that may appear in patient diary entries or self-reports. There is also emerging evidence that domain-specific pretraining improves NLP performance on mental health classification tasks compared to general models [72], though evidence of clinical outcome improvement in deployed therapeutic settings remains limited. These models therefore represent technically motivated starting points for exploration. For longitudinal or multimodal assessment, sequence models such as BiLSTMs (Bidirectional Long Short-Term Memory) [73,74] offer further possibilities for modelling temporal changes in symptoms and emotional states over time.

Grounded in these practitioner-identified needs, we suggest that a future design agenda could explore NLP-based approaches capable of contextual understanding of patient text. For instance, tools that can categorize language by emotional tone, cognitive content, or life domain, and link these to clinical patterns over time. Drawing inspiration from LIWC-22 (Linguistic Inquiry and Word Count) [75-77] in its approach to categorizing text according to linguistic and psychological dimensions, future systems could use contextual embeddings and machine learning to perform dynamic classification and thematic categorization of patient data, capturing richer nuances in language, emotion, and cognition. This suggestion is inspired by what practitioners described as missing from current tools, namely, the ability to go beyond surface-level keywords to detect patterns across symptom categories, emotional distress, social withdrawal, and risk factors. The integration of NLP tools for emotion and cognitive pattern analysis into HCI-driven mental health technologies also increases transparency [78] and supports more informed decision-making [79]. By linking AI outputs to specific text segments, practitioners can understand how patient data is processed and acted upon, supporting explainability and human-AI partnership. These design possibilities are directly inspired by practitioner concerns, and future co-design work is needed to assess their clinical feasibility.

### Co-Designing New Models of Trust in AI-Based Diagnosis Decisions

Given the high ethical sensitivity of diagnosis, we propose co-designing AI diagnostic support tools based on a human-AI partnership model, where AI serves as a validator of practitioners’ judgment rather than a replacement. This implication is grounded in our findings, where practitioners expressed resistance to autonomous AI diagnosis, citing the risks of dehumanization and oversimplification of complex clinical judgments. This approach directly addresses the principles of autonomy and nonmaleficence, particularly in depression care, where gender, age, and ethnic biases may be replicated in AI models. Practitioners’ concerns about AI diagnosis were not simply a matter of building trust in the technology: autonomy pointed to preserving practitioners’ decision-making authority, while nonmaleficence highlighted the risks of misdiagnosis. Together, these principles point toward a human-AI partnership model, rather than a more autonomous diagnostic system.

Previous HCI work has indeed indicated the harmful implications of automated diagnosis delivered without practitioners’ input or support [22,62]. We advocate for the human-AI partnership previously proposed through codesign of AI interfaces for CBT interventions [4]. We extend the value of such human-AI partnership within and across the entire therapeutic journey, from assessment and diagnosis to interventions, as well as to a broader range of therapeutic approaches including person-centered, psychodynamic, and humanistic therapy. In addition, we argue for the importance of a theoretical foundation for AI design for mental health. For this, a promising lens is medical ethics, which positions the technological advent of AI as a major risk for dehumanizing mental health care [39,42-45]. Medical ethics has also started to explore the challenges of hybrid decision-making and the need for alternative models of epistemic responsibility that such decisions entail [80]. More specifically, practitioners’ accounts highlight the importance of safeguarding their central role in overseeing AI-based decisions, and against the risk of emotional disconnect by maintaining the much-needed humanness [4] of therapeutic rapport [39]. Our study explored participants’ views on AI potential rather than actual trust in AI technologies. Future work is needed to investigate new models of trust that practitioners, as well as patients, are prepared to negotiate for AI-based technologies for mental health. Co-design studies emerging in this space [4] are promising. We can also look at candidate models of trust from the ethics of AI research area. One such model is that of discretionary authority through which practitioners decide to give AI authority for specific decisions, that is, to trust them, while remaining morally accountable for their implications [81]. Such models are particularly important for exploring the ethical concerns regarding ownership of misdiagnosis.

Practitioners expressed the view that when AI systems are designed with explainability, transparency, and practitioners’ oversight, they could enhance the practitioner-client alliance [82]. Our findings indicate that practitioners considered the importance of these qualities in the practical realities of clinical settings. They raised concerns about how sensitive data is stored and shared, and highlighted the need for clear, understandable rationales behind AI-generated recommendations. This included preferences for anonymized data handling, password-protected systems, and transparent documentation features. Practitioners also pointed to the value of interpretable outputs as a way to maintain control and ensure the technology aligns with ethical and patient-centered care.

### Designing LLM-Powered Personalized and Adaptive Mental Health Support Systems

To address practitioners’ needs for personalized, continuous support, we focus on the treatment stage, emphasizing interventions that extend beyond therapy sessions. Practitioners identified key challenges aligned with both beneficence and justice: the need for personalized, timely interventions (not generic recommendations) delivered when patients need them most. By considering beneficence and justice together, we can think beyond simply adding more AI features to a specific requirement for adaptive and personalized support, and also to ensure access for patients with different needs. These practitioner-expressed needs point toward novel design opportunities for AI systems capable of continuous, adaptive support, with LLMs representing one technically promising candidate.

To address these needs, we propose LLM-powered systems that continuously integrate multimodal patient data, including mood tracking, thought patterns, behavioral observations, and diary entries, to deliver personalized, adaptive CBT-informed interventions [54,83,84]. This approach leverages LLMs’ contextual understanding to (1) generate personalized interventions specific to each patient’s diagnosed condition and therapeutic approach; (2) adapt recommendations based on real-time mood and behavior changes; and (3) incorporate explainable AI to make reasoning transparent (addressing autonomy concerns). These 3 capabilities map directly onto specific practitioner concerns identified in our findings: personalization responds to practitioners’ calls for detailed, individual-specific treatment plans rather than generic recommendations; real-time adaptation addresses their emphasis on monitoring symptom changes and risk factors between sessions; and explainability directly responds to practitioners’ stated need to understand how AI reaches its recommendations and to maintain oversight of AI-generated decisions.

By integrating this system within a human-AI partnership framework, where practitioners retain oversight and can adjust AI recommendations, we frame treatment as an ongoing dialogue rather than autonomous decision-making. This could also incorporate proactive risk detection to flag deterioration between sessions, supported by practitioners’ recognition that carefully designed AI has a valuable role in monitoring clients in crisis [85].

### Limitations

Our study has several limitations that future work should address. First, our sample of 18 counselors and psychotherapists is relatively small, limited to the UK context, and drawn from a single professional association, which may limit the diversity of perspectives represented. The sample size reflects the practical constraints of recruiting specialist practitioners and is consistent with the exploratory nature of such qualitative research. Nevertheless, our purposive sampling focused on recruiting expert mental health practitioners, ensuring that our participants were able to draw from their extended clinical practice to reflect on the preuse acceptability of AI technologies. Thus, aligned with previous findings on the value of smaller homogeneous samples to reach meaning saturation [86], we also argue that our homogeneous sample of 18 participants has provided information-rich accounts for an in-depth analysis. We also acknowledge the gender imbalance of our sample; yet this is indicative of the feminization of the health care profession, including mental health practice [87].

Second, the scenario presented on AI for depression detection was designed to increase practitioners’ AI literacy, as few had experience using AI in their clinical practice. Improving AI literacy among mental health practitioners before engaging them in research or implementation is considered important, as limited training and understanding have been identified as key barriers to AI adoption in clinical settings [4,14,88]. The AI presentation at the start of our interviews was therefore a deliberate methodological choice to ensure meaningful participation. We also acknowledge that it may have anchored participants’ responses around our provided examples of AI use, potentially impacting the envisaged benefits and risks. It is important to note that while the scenario was specific to depression detection, the interview questions were intentionally broader, covering AI use across all 3 therapeutic stages of assessment, diagnosis, and treatment, thus allowing participants to draw on their wider clinical experience beyond the scenario presented. Future studies could use multiple scenarios or open-ended prompts to further reduce this potential bias. Finally, although multimodal data can enhance AI predictions, practical challenges in collecting and integrating such data in clinical settings were not fully explored. Despite these limitations, the findings provide valuable insights into practitioner perspectives on AI integration in mental health care.

### Conclusions

This study explored mental health practitioners’ perspectives on AI technologies across the main stages of therapeutic practice: assessment, diagnosis, and treatment. Through interviews with 18 practitioners, we found preliminary evidence suggesting that AI may hold different value and raise different ethical concerns depending on the therapeutic stage, from the perspective of this group of UK practitioners. Practitioners see the most potential for AI in treatment but show strong resistance to autonomous AI diagnosis. Practitioners expressed the view that AI ought to validate and enhance their clinical judgment rather than make independent decisions. This human-AI partnership model emerged as essential across all therapeutic stages. We organized the ethical challenges practitioners identified using 4 biomedical principles: nonmaleficence, beneficence, justice, and autonomy. Key concerns include the validity of AI decisions based on limited data, the need for inclusive and accessible technologies, privacy and security of sensitive patient information, and transparency in how AI reaches its recommendations. Based on these findings, we proposed four design directions for AI technologies in mental health: (1) clinical assessment hubs that integrate multimodal patient data for transparent, ongoing assessment; (2) NLP-AI tools to capture deeper psychological meaning in patient language; (3) co-designed models of trust that position AI as a validator of practitioners’ diagnostic judgment rather than an autonomous decision-maker; and (4) LLM-powered personalized support systems that deliver adaptive interventions while maintaining human oversight and practitioners’ central role.

## Appendix

Multimedia Appendix 1 Interview guide and questions used in the study.

## Abbreviations

BiLSTM: Bidirectional Long Short-Term Memory
CBT: cognitive behavioral therapy
HCI: human-computer interaction
LIWC: Linguistic Inquiry and Word Count
LLM: large language model
NLP: natural language processing
