# Supplementary material for: “There is a Place for it”—Practitioners’ Perspectives on Situated Ethical Acceptability of AI Technologies for Mental Health Practice: Qualitative Study
Source: JMIR Ment Health. 2026 Oct 19;13:e91884. doi: 10.2196/91884 (PMC13728769; doi:10.2196/91884)
Supplement: Supplementary file 1 [file mental_v13i1e91884_app1.docx]

***Faculty of Science and Technology Research Ethics Committee (FSTREC)***

***Lancaster University***

# Draft of Interview Guide

This semi-structured interview guide was used to explore practitioners' views on AI across therapeutic stages (Part A) and their anticipated ethical concerns (Part B). As the interviews were semi-structured, not all questions were asked verbatim or in the same order for every participant, and follow-up questions were used to explore participants' responses in more depth.

- **DEMOGRAPHICS**:

1. How long have you been working in your practice to support mental health?
2. What is your formal education, including highest level of education?
3. Do you have any additional training for mental health?
4. What is your age?
5. What is your gender identity?

**scenario:**

Imagine an AI system integrated into your clinical practice that analyses patient input, such as written entries, spoken language during sessions, or responses to digital check-ins, to detect symptoms of depression. The system looks for patterns such as negative language, low mood, hopelessness, and social withdrawal. Based on this analysis, the system assesses the patient's risk level and can offer personalized intervention suggestions, such as Cognitive Behavioural Therapy (CBT) techniques or mindfulness exercises, tailored to the individual's reported symptoms and needs.

**A- Practitioners' Views on AI Across Therapeutic Stages**

1. How do you see AI used to support mental health practice? Can you provide examples of specific types of support at each stage?
2. Would you use a tool that can help to personalise an intervention/assessment/diagnosis based on an individual’s captured data? How would you envision the implementation of an AI-based mental health technology in your practice?
3. Are there any specific features or functionalities you would like to see in an AI-based mental health technology? Which ones? Why? Which stage?
4. How do you think this AI can enhance clinicians' understanding of patients' mental health and?
5. Can you provide examples of how it could benefit clinical practice across all the stages?
6. How important do you believe personalised interventions are for individuals with depression? How could this AI contribute to tailoring treatment / Assessment / Diagnosis?
7. What challenges do you foresee in implementing AI into existing mental health practices? How can potential issues like data privacy and security be addressed?
8. How could the AI positively impact treatment strategies/ identifying symptoms/ assessment/ diagnosis? Are there any concerns or limitations?
9. What support or resources do you think would be valuable for clinicians using AI? Are there any additional features or functionalities that could enhance its effectiveness? Which stage?
10. How should the AI be used in clinical practice to ensure it supports clinicians in comprehending the patient's mental state?
11. Are there any ethical considerations or potential risks associated with using AI in mental health assessments/ diagnosis and interventions? How can these concerns be addressed responsibly?
12. Based on your expertise, do you have any suggestions or recommendations? Are there any areas or aspects that require additional attention or exploration?

**B-**  **ETHICAL DESIGN CONSIDERATIONS:**

1. Can you think of any benefits of AI-based mental health technologies? Please explain.
2. Based on your expertise, what are the main ethical challenges of AI-based mental health technologies?
3. Can you envisage ways in which these can be overcome? What aspects should we consider when we design AI-based mental health technologies?
4. What factors should be considered to ensure that AI-based interventions are culturally sensitive and inclusive for diverse populations?
